# Hospital pharmacists’ experiences of participating in a partnered pharmacist medication charting credentialing program: a qualitative study

**DOI:** 10.1186/s12913-021-06267-w

**Published:** 2021-03-19

**Authors:** Hannah Beks, Kevin Mc Namara, Elizabeth Manias, Andrew Dalton, Erica Tong, Michael Dooley

**Affiliations:** 1 School of Medicine, Deakin University, Geelong, Victoria Australia; 2 School of Nursing and Midwifery, Deakin University, Burwood, Australia; 3 School of Health and Social Development, Deakin University, Burwood, Australia; 4 Alfred Health, Prahran, Victoria Australia

**Keywords:** Pharmacy, Health services Research, Medication therapy management, Patient care management, Patient safety, Multi-disciplinary, Education, pharmacy

## Abstract

**Background:**

Medication-related errors are one of the most frequently reported incidents in hospitals. With the aim of reducing the medication error rate, a Partnered Pharmacist Medication Charting (PPMC) model was trialled in seven Australian hospitals from 2016 to 2017. Participating pharmacists completed a credentialing program to equip them with skills to participate in the trial as a medication-charting pharmacist. Skills included obtaining a comprehensive medication history to chart pre-admission medications in collaboration with an admitting medical officer. The program involved both theoretical and practical components to assess the competency of pharmacists.

**Methods:**

A qualitative evaluation of the multi-site PPMC implementation trial was undertaken. Pharmacists and key informants involved in the trial participated in an interview or focus group session to share their experiences and attitudes regarding the PPMC credentialing program. An interview schedule was used to guide sessions. Transcripts were analysed using a pragmatic inductive-deductive thematic approach.

**Results:**

A total of 125 participants were involved in interviews or focus groups during early and late implementation data collection periods. Three themes pertaining to the PPMC credentialing program were identified: (1) credentialing as an upskilling opportunity, (2) identifying the essential components of credentialing, and (3) implementing and sustaining the PPMC credentialing program.

**Conclusions:**

The PPMC credentialing program provided pharmacists with an opportunity to expand their scope of practice and consolidate clinical knowledge. Local adaptations to the PPMC credentialing program enabled pharmacists to meet the varying needs and capacities of hospitals, including the policies and procedures of different clinical settings. These findings highlight key issues to consider when implementation a credentialing program for pharmacists in the hospital setting.

**Supplementary Information:**

The online version contains supplementary material available at 10.1186/s12913-021-06267-w.

## Background

Medication-related errors are one of the most frequently reported clinical incidents in Australian hospitals [1]. For every three patients admitted to hospital, approximately two medication errors occur during admission [1]. Transitions of care are also problematic. Research has found that a majority of hospital discharge summaries that have not had a pharmacist involved, have had at least one medication error [2]. Medication errors are a major safety risk and can result in harm to patients. Older patients with multiple co-morbidities are at a heightened risk for experiencing medication-related harm, particularly in the acute care setting [3, 4]. At an international level, medication errors also pose a significant financial burden on health care systems, resulting in prolonged hospital stays and increased patient morbidity and mortality [5, 6].

Human error is a major contributor to the occurrence of medication errors within the hospital setting. Factors contributing to human error have been categorised in the literature as active failures (e.g. lapses and a failure to adhere to rules), error-producing conditions (e.g. in the work-environment, within the team, at the individual level, task level or patient level) and latent conditions (e.g. organisational training processes, systems and cultural dynamics around medications) [7–9]. Individual behaviours of clinicians also play an important role in interacting with these factors when prescribing medications [10]. For example, prescribers may have different ways of managing error-producing conditions (e.g. a high patient load) and prioritise prescribing critical medications (e.g. anticoagulants) which may lead to the omission of other medications [10]. Likewise, clinician behaviour also mediates with these factors in the administration of medications [11]. For example, incorrect administration techniques, interruptions and mismanagement of time have been identified as factors contributing to medication administration errors by nurses [11].

Interventions increasing the role of pharmacists in medication management are key to mitigating the incidence of medication errors and related adverse events in the health care system [12, 13]. Credentialing programs to expand the scope of practice of pharmacists have been explored as a potential strategy to increase the involvement of pharmacists caring for patients and thereby improve medication safety [14, 15]. In the health care context, credentialing programs are often used to educate and assess the competency of health professionals in advanced clinical practice situations [14]. Health professionals who have attained a credentialed status can support their colleagues in clinical practice as a means of improving the safety and quality of care for patients.

A Partnered Pharmacist Medication Charting (PPMC) model was developed to increase the involvement of credentialed pharmacists in clinical decision making relating to medication management for acute care patients being admitted under a general medicine team [16]. The PPMC model involved pharmacists leading the medication reconciliation and charting process for patients who were being admitted under a general medicine unit. They also generated a medication plan for the admission, in consultation with the admitting doctor. In 2012, a significant reduction in rates of medication errors were demonstrated though an initial single-site unblinded Randomised Controlled Trial (RCT) of the PPMC model [16, 17].

A subsequent multi-site implementation trial of the PPMC model was conducted across seven Australian hospitals over a period of twelve months from 2016 to 2017, and involved data collection from a total of 8648 patients [18]. As part of this model, eligible pharmacists were required to complete a PPMC credentialing program to upskill to a PPMC model of clinical practice. Results from the multi-site implementation trial found that patients who received the PPMC model had a reduced median length of inpatient hospital stay (4.7 days to 4.2 days) compared to those who received standard care [19]. Furthermore, fewer medication charting errors were found in patients who received the PPMC model compared to those who received standard care (0.5% versus 19.2%) [18]. As the model was found to provide both overall cost-savings and improved health outcomes, it was also deemed to be highly cost-effective [19].

In the literature, there are a few examples of credentialing programs implemented for pharmacists. One such example included a pilot of a national pharmacy-credentialing program for advanced practice pharmacists in Australia [15, 20]. This credentialing program involved the submission of a practice portfolio detailing clinical experience, competencies and completed postgraduate study [15]. In other countries, pharmacy credentialing programs have been developed to upskill pharmacists in pain management for palliative care patients and in advanced practice skills in the critical care context [14, 21]. Little is documented about the experiences and attitudes of pharmacists who have completed a pharmacy credentialing program, particularly in the inpatient hospital setting [22]. Examining the experience and attitudes of pharmacists completing a credentialing program in the acute general medicine inpatient context through qualitative inquiry, is imperative to improving the implementation of credentialing programs. The COnsolidated criteria for REporting Qualitative studies (COREQ) has been used to guide the reporting of this study (Supplementary File 1) [23].

## Aim of the study

To explore the experiences of pharmacists and key informants involved in delivering or participating in the PPMC credentialing program and examine perceptions of how the objectives of the program were met.

## Ethics approval

Ethical approval was obtained from Alfred Health (Project No. HREC/16/Alfred/40).

## Methods

### Study design

A qualitative study design using interviews and focus group sessions was undertaken to evaluate the PPMC implementation trial and examine the experiences and perceptions of participants in relation to delivering and participating in the PPMC program [24]. Qualitative data were collected at two time points, early implementation (June to September 2016) and late implementation (July to December 2017).

### Setting and intervention

The PPMC credentialing program was delivered to seven participating hospitals (metropolitan *n* = 5, regional *n* = 2) implementing the PPMC model within their general medicine unit and Emergency Department (ED). Further detail around the PPMC model has been published elsewhere [18]. The PPMC pharmacist credentialing program involved prescribed readings, an online module, supervised clinical case studies and an Objective Structured Clinical Examination (OSCE) (Table 1).

**Table 1:** PPMC-credentialing program structure

Credentialing component	Mode of delivery	Time to complete
Prescribed readings	Online	One hour
Online module	Online	One hour
Supervised clinical case studies	Face to face	Five to ten hours
Objective Structured Clinical Examination	Face to face	One hour

A senior pharmacist and consultant general physician from each hospital received training from the co-ordinating study site to deliver the face-to-face components of the credentialing program, which included case studies and the final OSCE. The content of the training, case studies and OSCE were determined by the co-ordinating study site and consistent across all included sites. The OSCE involved an assessment of two cases by a consultant general physician and a senior pharmacist.

Eligible pharmacists were those with at least 2 years of clinical experience in a hospital and 6 months experience managing complex medical patients. This provision was to ensure that participating pharmacists were competent in managing complex general medical patients and able to expand their clinical scope of practice to that of a general medicine PPMC credentialed pharmacist. Competency was determined by a senior clinician who assessed participating pharmacists’ ability to obtain a comprehensive medication history from a patient, develop a medication management plan in collaboration with an admitting medical officer and chart medications on the inpatient medication chart.

### Study recruitment

Key informants (directors of pharmacy departments, nursing and medicine) and pharmacists engaged with the PPMC trial (including credentialed and non-credentialed pharmacists), were identified by a project officer from each site. Project officers were pharmacists who were funded to coordinate the trial at each hospital. Key informants directly involved in the delivery of the PPMC program, and pharmacists who participated in the PPMC credentialing program or who were preparing to participate in the PPMC credentialing program, were purposively sampled by researchers for their experience in relation to the phenomena of interest and invited to a focus group or interview. Project officers assisted researchers, who were external to participating hospitals, with coordinating interview and focus group session times with participants.

### Data collection

An exploratory approach was used to conduct interviews and focus group sessions. This approach was necessary for the purpose of examining the experiences and perceptions of pharmacists and key informants involved in implementation of the PPMC model. Interviews were undertaken with key informants to gain rich insights into the implementation of the PPMC credentialing program, whereas focus groups were conducted with groups of participating pharmacists to examine both collective and individual experiences of program participation. A guide for interviews and focus groups was developed (Supplementary File 2), which examined key concepts within the Victorian Innovation and Reform Impact Assessment Framework (VIRIAF) domains of effectiveness, efficiency and sustainability [25]. These included safety and quality of care, workforce capacity, workforce satisfaction, efficiency inputs and outputs and enablers and barriers for sustainability. Using VIRIAF as an evaluation framework was recommended by the evaluation advisory group as it was the formal framework for program impact assessment adopted by the funding body. Although the same guide was used for both interviews and focus groups, more time was allowed during focus groups for participants to prompt and interact with each other as part of examining the collective experience of participating in the PPMC credentialing program.

A Plain Language Statement (PLS) was provided to participants by the researchers at the beginning of each session which provided information around the purpose of the research, what participants could expect from participating in the research and detail around the voluntary nature of participation, including right to withdraw from research. Written informed consent was obtained by researchers from participants prior to the conduct of interviews and focus groups. All researchers conducting interviews (HB, KM, EM) had experience in undertaking qualitative research (experience ranged from undertaking at least one qualitative research project to multiple qualitative research projects using different methodologies) and had clinical backgrounds (pharmacy and nursing). Researchers involved in the collection and analysis of qualitative data, did not work clinically at the participating sites. After interviews and focus group sessions, researchers engaged in an informal debrief which served as a platform to reflect on sessions and consider the impact of researcher bias on the sessions as per reflexive practice [26]. Interviews and focus group sessions were held at participating hospitals and in person, with some key informant interviews conducted over the telephone. Sessions were approximately 1 hour in duration, were audio-recorded and transcribed verbatim. A copy of the de-identified transcript was provided to participants on request (member checking), with the opportunity to offer further feedback to researchers.

### Data analysis

De-identified qualitative transcripts were imported into QSR NVivo for Windows, version 11 (QSR International Pty Ltd., Melbourne, Vic, AU) for analysis [27]. A pragmatic approach to thematic analysis was used to identify the key actions, processes and issues pertaining to the PPMC credentialing program [28, 29]. This involved initial open coding of transcripts (an inductive approach) and use of the VIRIAF evaluation framework domains to broadly categorise codes and emerging concepts (a deductive approach) [25, 30]. This was followed by second cycling coding methods (axial coding) to compare and contrast emerging concepts [25, 30]. Two researchers were involved in developing a coding guide after independently reviewing several transcripts. This was then reviewed by a third researcher where any discrepancies were discussed, leading to a thematic analysis of key concepts pertinent to the PPMC credentialing program across the VIRIAF domains [28, 31]. Themes were developed through discussion between the three researchers and through an iterative process of reviewing the coding guides [29].

### Rigor of qualitative methods

Strategies to improve the rigor of qualitative methods used were guided by Lincoln and Guba’s (1985) criteria of trustworthiness and authenticity in qualitative research, comprising credibility, transferability, dependability and confirmability [32]. Strategies to improve and test the credibility of findings included a prolonged engagement with participants evidenced by two periods of data collection (early implementation and late implementation) which provided researchers with an opportunity to test and examine themes developed, and use of member checking. To improve dependability and confirmability, researchers provided a detailed description of the coding process and development of themes, and engaged in frequent informal debriefs and reflexive practice. Purposive sampling was employed to improve the transferability of findings to other health services and context.

## Results

### Interviews and focus group demographics

A total sample of 125 participants were involved during the early implementation (interviews *n* = 5, focus group sessions *n* = 11) and late implementation (interviews *n* = 19, focus group sessions n = 11) qualitative data collection periods as part of the broader evaluation. Focus groups ranged from having two to twelve participants per session. A total of 50 h of audio recordings were transcribed verbatim. Of the 125 participants involved in early implementation and late implementation data collection, 64 participants were pharmacists with various years of clinical practice (Table 2) and 14 were key informants (Table 3), with data included in this study focusing on the PPMC credentialing program. Of the 64 pharmacist participants, 13 participants were involved in both the early and late implementation data collection periods.

**Table 2:** Pharmacist demographics

	Early implementation (*n* = 32)	Late implementation (n = 32)
Female: male ratio	27:5	23:8 (1 unknown)
Age range in years (mean)	23–60 (30.5)	24–54 (30.7)
Years in profession range (median)	1–39 (6.0)	1.5–30 (6.0)
Years working in the Australian hospital system range (median)	1–39 (6.0)	1.5–30 (5.5)
Proportion of participants PPMC credentialed (%)	*n* = 24 (75%)	*n* = 21 (66%)

**Table 3:** Key informant demographics

	Late implementation (*n* = 14)
Female: male ratio	6:8
Age range in years (mean)	32–68 (46.3)
Years in profession range (median)	7–40 (20)
Years working in the Australian hospital system range (median)	3–40 (20.5)
Medical consultants	5
Directors of pharmacy	5
Directors of other departments (e.g. nursing, clinical services)	4

Three themes pertaining to the PPMC credentialing program were developed through data analysis: (1) credentialing as an upskilling opportunity, (2) identifying the essential components of credentialing, and (3) implementing and sustaining the PPMC credentialing program.

### Credentialing as an upskilling opportunity

General satisfaction with the PPMC credentialing program was communicated by credentialed pharmacists, senior pharmacists and key informants involved in the implementation trial. Credentialing provided pharmacists with an opportunity to upskill, consolidate clinical knowledge, increase clinical confidence and expand their scope of practice. Most participants were satisfied with the components of the program and conveyed that the program equipped them with the skills necessary to undertake the PPMC pharmacist role.

“I think it’s being confident in your knowledge as well … I think this is where credentialing helps, to be able to make [a] formulated plan, make a decision and then present that to the doctor, rather than deciding whether what the doctor did was appropriate or not.”

Early implementation focus group, credentialed pharmacist.

Some participants identified limitations of the program in preparing pharmacists for the PPMC pharmacist role. These limitations largely related to how being assessed for PPMC competency through an OSCE, a simulation exercise, differed to undertaking the PPMC role in real time in a clinical setting. For example, one participant discussed how the interaction between a pharmacist and a doctor during the OSCE, differed to interactions occurring in clinical practice where contextual factors such as time pressures and team dynamics were at play. Another participant stated that there were more resources available to assist the pharmacist with making clinical decisions in practice, than there were during the OSCE.

“I think people would need to be made aware that the model of the conversation that you have during the practice cases and the OSCE is going to be different from what how you end up having the conversation in real life.”

Early implementation focus group, credentialed pharmacist.

Aside from performing the PPMC role in collaboration with a doctor, credentialed pharmacists were able to identify positive flow-on effects to their clinical practice as a result of participating in the PPMC credentialing program. These included improving the process of obtaining a medication history and ability to think critically about medications.

“I didn’t always make the connections between medications and their indications. So, making sure the indication always matched up … if a medication was there that didn’t have an indication, now it’s something I would always question …“.

Late implementation interview, credentialed pharmacist.

Senior credentialing pharmacists observed that the PPMC credentialing process was also useful for identifying clinical knowledge gaps in participating pharmacists within their hospital. A benefit of this process was the potential for credentialing programs to improve the practice of pharmacists in general. Examples included conducting a more comprehensive patient interview and being able to rationalise medications in order to make suggestions to the doctor about medication regimes.

“From a credentialing point of view, the impact it’s had on me is that it gives me an opportunity to kind of allow pharmacists to see gaps in their knowledge and practice. So it’s a tool that I use to upskill pharmacists to enable them to see better ways of doing things …“

Late implementation focus group, senior credentialing pharmacist.

### Identifying the essential components of credentialing

All components of the PPMC credentialing program were considered to be of benefit to the clinical practice of participating pharmacists and imperative to the implementation of the PPMC model. Participants identified that the credentialing program components equipped pharmacists with the skills to implement the PPMC model. However, some components were deemed more relevant or useful than others and there was some variation in participant attitudes to the PPMC credentialing program across the seven hospitals. For example, some pharmacists completing the PPMC credentialing program had difficulty with the online Multiple Choice Quiz (MCQ) due to a lack of knowledge regarding prescribing protocols and legal regulations, whereas others had no issues. Strong support was also offered for the use of case studies in preparing participants for the OSCE, which was a mandatory component of the PPMC credentialing program. In particular, there was a preference for complex clinical cases that demanded consideration of multiple patient factors.

“ … so you could just have cases that, like it doesn’t have to be a real case, like it could just be something with a lot of issues that you need to talk, or a lot of considerations that you need to speak to the prescriber about … medications related to the other things, so, like interactions, bloods, I guess patient considerations as well.”

Early implementation focus group, credentialed pharmacist.

Observing peers engage with the PPMC credentialing process was also valued and offered additional learning opportunities for participants. The opportunity to practise the PPMC process with the supervision of senior pharmacists, provided a forum for participants to refine their clinical skills and to learn from observing peers in a structured manner, which was generally regarded as a new approach to clinical education for participating pharmacists.

“It was good to see how other people interacted with the registrar and undertook their medication review on admission and then, and then to carry it out yourself and have that discussion with the supervising pharmacist for the first, certain amount of times that we’re meant to do, it was good.”

Late implementation focus group, credentialed pharmacist.

Pharmacists could also reflect that the structured clinical conversation assessed during the OSCE did not always translate into clinical practice because the assessment did not involve an interaction in real time within the clinical setting. Some participants advised that it would be beneficial to complete the PPMC credentialing OSCE using real patients in the clinical setting.

“ … it’s not just about my pharmacist passing the OSCE, it’s about them doing it in real time … making sure they actually reconcile the bits that they need to but then can also condense what they’re saying to have a very short conversation with the prescribers.”

Early implementation focus group, senior credentialing pharmacist.

In reflecting on the components of the credentialing program, participants acknowledged the need for a focus on preparing PPMC credentialed pharmacist for developing a medication management plan for complex patients and for those with multiple chronic conditions. This view was considered of particular relevance to the acute general medicine clinical setting.

“… the training probably needs to include a couple more scenarios where you might have an unclear diagnosis and you’ve got to treat for multiple different diagnosis depending on what the issues are.”

Late implementation interview, credentialed pharmacist.

### Implementing and sustaining the PPMC credentialing program

Requirements for sustaining and expanding the credentialing program were discussed mostly by key informants (e.g. directors of departments). Ensuring that the credentialing process could be adapted to meet the needs of local health services, was a key recommendation. Maintaining the fidelity of the credentialing program, as implemented during the trial, was considered to be particularly challenging due to high staff turnover, the need to backfill staff, resourcing constraints and pharmacy rotations. It was identified that credentialed pharmacists were rotated through hospitals and clinical specialities, which required an ongoing PPMC credentialing program for new pharmacists. This was problematic when the time required from senior medical and pharmacy staff to credential pharmacists was regarded as unsustainable as senior staff often had multiple roles in hospitals and were not always available to oversee the credentialing process.

“So the other component is really credentialing the pharmacists which is a very time consuming process … It’s creating that capability within this health service to be able to credential our own pharmacists.”

Late implementation interview, key informant.

Maintaining the consistency of the PPMC model across hospitals through standardisation of the PPMC credentialing program after the completion of the trial, was also discussed. A few participants suggested that embedding the PPMC credentialing program in undergraduate or postgraduate courses was a potential solution. The need for the national standardisation of pharmacy credentialing programs was also highlighted. Standardising the clinical practice of pharmacists was understood to be a necessary step to ensure PPMC credentialed pharmacists were not disadvantaged when rotating through other hospitals that did not have a PPMC model of care.

“My only main issue is that these sort of localised credentialing arrangements … I think are not ideal because I think they disadvantage some people in places. Ideally, you’d want a broader recognised qualification.”

Late implementation interview, key informant.

In transferring the PPMC model to other clinical settings (i.e. surgical, oncology), participants identified the need to modify the PPMC credentialing program content to reflect the clinical requirements of other specialties. Integrating specialty specific case studies and accommodating for other factors impacting the PPMC process such as perceived reduced access to senior clinicians for the clinical conversation in a surgical unit were also discussed.

“… the process is the same regardless of who is involved … from having the conversation about the plan, training for the medical staff, I think that’s where we’re going to have to think carefully about how we, how we customise that depending on which people are in … who the pharmacist, particularly in the surgical stream, who the pharmacists are engaging with.”

Late implementation interview, key informant.

## Discussion

Findings support the implementation of a credentialing program for pharmacists engaging in a PPMC model of practice across multiple hospitals. The PPMC model has been shown to reduce the length of inpatient hospital stay and to be highly cost-effective [19]. The PPMC credentialing program was considered very beneficial for expanding the competencies and the clinical scope of practice for pharmacists in the acute general medicine setting – a key component of effectiveness stated in the VIRIAF [25]. Essential components of a PPMC credentialing program were identified, which included the importance of peer learning and opportunities to practice skills in real time. Other program inputs were also identified which were able to be mapped to the VIRIAF domain of efficiency [25]. Implications for implementing the PPMC credentialing program in other contexts and sustaining the credentialing program were identified and included resourcing and specialty-specific requirements. These implications expanded upon barriers and enablers for the sustainability of workforce interventions identified by VIRIAF [25], which included ongoing supervision requirements, incorporating the workforce project into standard practice and workforce mix.

Although there is a paucity of research around credentialing programs for pharmacists and health professionals in general, there is strong support for expanding pharmacy scope of practice to ensure the efficient utilisation of pharmacy clinical skills in an era of increasing demand for healthcare [14, 33]. Credentialing programs also have potential in developing pathways to advanced practice roles for pharmacists across a variety of clinical settings [14, 15, 34]. Qualitative evidence supporting general satisfaction with the PPMC credentialing program, highlights how such programs enable pharmacists to expand their clinical skill set and become more equipped to advocate for medication safety in the acute care setting.

A key identified strength of the PPMC credentialing program was the use of supervised clinical case studies and an assessment of clinical competence (e.g. OSCE), components included in other inter-disciplinary health credentialing programs [35]. In other health professions, such as nursing and medicine, experiential learning opportunities through simulation-based exercises have proved effective in increasing clinical confidence, skills and self-efficacy [36, 37]. Developing the PPMC credentialing program to incorporate elements of high fidelity clinical simulation (e.g. assessment of interactions between doctors, pharmacist and patients in real time), may ensure participants are more adequately equipped with the skills necessary to undertake the PPMC pharmacy role. For example, this could involve conducting the OSCE in a clinical setting (e.g. emergency department) with real patients or a patient simulator (e.g. using Human Patient Simulator (HPS) technology) [38]. In undergraduate pharmacy education, high fidelity patient simulation using a HPS, has proven to be more effective in improving knowledge acquisition, retention and decision-making capacities, when compared to the use of case-based learning (CBL) [38].

A key challenge for the sustainability of the PPMC credentialing program, and PPMC model more broadly, is ensuring that the implementation of the PPMC credentialing process is consistent in hospitals following the completion of the trial. Ensuring hospitals are able to allocate the required resources and staffing to the PPMC credentialing program has the potential to be problematic and dependent on the prioritisation of the PPMC model within each hospital. Like other pharmacy programs, the PPMC credentialing program needs to be adapted to meet the needs of local hospitals [39]. This adaptation includes meeting the requirements of hospital-specific protocols in addition to state-wide guidelines. This highlights an additional challenge of ensuring the consistent implementation of the PPMC credentialing program in other hospitals who are new to a PPMC model of health care. Furthermore, transferring the PPMC model from general medicine to other clinical specialities will require modification of the clinical content of the program. This could involve the use of specialty specific case studies addressing medication management issues pertinent to the specialty context.

Although this qualitative study examined the experiences and perceptions of pharmacists and key informants involved with the PPMC credentialing program, the findings are of value to hospitals and decision-makers considering implementing other pharmacy credentialing programs. For example, this transference could include examining key issues mediating the sustainability of other credentialing programs. Some limitations of the study include combining the analysis of data from interviews and focus groups to develop themes, rather than analysing interviews and focus groups separately to examine any differences attributed to the data collection method used. Further research is required around the effectiveness of pharmacy credentialing programs more broadly, and the adaptations required to meet the needs of hospitals and clinical specialties. At a policy level, such research would be beneficial in informing the standardisation of an expanded scope of clinical practice for pharmacists (both nationally and internationally), thereby advocating for the credibility of the profession in improving the safety of medication management.

## Conclusion

The evaluation of the PPMC credentialing program supports the role of credentialing programs in the ongoing professional development and upskilling of pharmacists to expanded practice roles. Further research is required to evaluate the adaptation of the PPMC credentialing program to other clinical specialities and determine the sustainability of the program across other hospitals and specialties. At a policy level, consideration should be given for the standardisation of pharmacy credentialing programs to ensure the consistency and longevity of programs across hospitals.

## Supplementary Information

**Additional file 1: Supplementary File 1**. Consolidated criteria for reporting qualitative research (COREQ) checklist

**Additional file 2: Supplementary File 2**. Interview guide for health professionals and key informants

## Data Availability

The data that supports the findings of this study are stored at Deakin University. Restrictions apply to the availability of this data, which were used for the current study, and so are not publicly available.

## Abbreviations

CBL: Case-based Learning
COREQ: Consolidated criteria for reporting qualitative research
ED: Emergency Department
HPS: Human Patient Simulator
MCQ: Multiple Choice Quiz
OSCE: Objective Structured Clinical Examination
PLS: Plain Language Statement
PPMC: Partnered Pharmacist Medication Charting
RCT: Randomised Controlled Trial
VIRIAF: Victorian Innovation and Reform Impact Assessment Framework
VTE: Venous Thromboembolism
