# Supplementary material for: Hospital pharmacists’ experiences of participating in a partnered pharmacist medication charting credentialing program: a qualitative study
Source: BMC Health Serv Res. 2021 Mar 19;21:251. doi: 10.1186/s12913-021-06267-w (PMC7980669; doi:10.1186/s12913-021-06267-w)
Supplement: Supplementary file 1 — Additional file 1: Supplementary File 1. Consolidated criteria for reporting qualitative research (COREQ) checklist [file 12913_2021_6267_MOESM1_ESM.docx]

**Supplementary File 1. Consolidated criteria for reporting qualitative research (COREQ) checklist**

| **COREQ item** | **Page # reported/Not applicable (with reason provided)** |
| --- | --- |
| **Domain 1: Research team and reflexivity** | |
| Personal Characteristics | |
| 1. Interviewer/facilitator Which author/s conducted the interview or focus group? | Methods, para 1, p. 9 |
| 2. Credentials What were the researcher’s credentials? E.g. PhD, MD | Included in submission application form. |
| 3. Occupation What was their occupation at the time of the study? | Included in submission application form. |
| 4. Gender Was the researcher male or female? | Not applicable to study purpose. |
| 5. Experience and training What experience or training did the researcher have? | Methods, para 1, p. 9 |
| Relationship with participants | |
| 6. Relationship established Was a relationship established prior to study commencement? | Not applicable. No prior relationship with participants. |
| Participant knowledge of the  interviewer | |
| 7. What did the participants know about the researcher? e.g. personal goals, reasons for doing the  research | Methods, para 1, p. 9 |
| Interviewer characteristics | |
| 8. What characteristics were reported about the interviewer/facilitator? e.g. Bias, assumptions,  reasons and interests in the research topic | Methods, para 1, p. 9 |
| **Domain 2: study design** | |
| Theoretical framework | |
| What methodological orientation was stated to underpin the study? e.g. grounded theory,  discourse analysis, ethnography, phenomenology, content analysis | Methods, para 1, p. 7 |
| 10. Sampling How were participants selected? e.g. purposive, convenience, consecutive, snowball | Methods, para 3, p.8 |
| 11. Method of approach How were participants approached? e.g. face-to-face, telephone, mail, email | Methods, para 3, p.8 |
| 12. Sample size How many participants were in the study? | Results, para 3, p. 9-10 |
| 13. Non-participation How many people refused to participate or dropped out? Reasons? | Not applicable, no participants refused to participate. |
| Setting | |
| 14. Setting of data collection Where was the data collected? e.g. home, clinic, workplace | Methods, para 1, p. 9 |
| 15. Presence of non-participants Was anyone else present besides the participants and researchers? | Not applicable, no one else present. |
| 16. Description of sample What are the important characteristics of the sample? e.g. demographic data, date | Results, para 3, p. 9-10 |
| Data collection | |
| 17. Interview guide Were questions, prompts, guides provided by the authors? Was it pilot tested? | Supplementary file 1. |
| 18. Repeat interviews Were repeat interviews carried out? If yes, how many? | Results, table 2, p. 10 |
| 19. Audio/visual recording Did the research use audio or visual recording to collect the data? | Methods, para 1, p. 9 |
| 20. Field notes Were field notes made during and/or after the interview or focus group? | Not applicable, field notes were not made. |
| 21. Duration What was the duration of the interviews or focus group? | Methods, para 1, p. 9 |
| 22. Data saturation Was data saturation discussed? | Not applicable, data saturation was not the objective of qualitative approach as an exploratory descriptive design was used to identify key actions, processes and issues pertaining to the PPMC credentialing program (p. 9). |
| 23. Transcripts returned Were transcripts returned to participants for comment and/or correction? | Methods, para 1, p. 9 |
| **Domain 3: analysis and findings** | |
| Data analysis | |
| 24. Number of data coders How many data coders coded the data? | Methods, para 2, p. 9 |
| 25. Description of the coding tree Did authors provide a description of the coding tree? | Methods, para 2, p. 9 |
| 26. Derivation of themes Were themes identified in advance or derived from the data? | Methods, para 2, p. 9 |
| 27. Software What software, if applicable, was used to manage the data? | Methods, para 2, p. 9 |
| 28. Participant checking Did participants provide feedback on the findings? | Not applicable, participants did not provide feedback on findings. |
| Reporting | |
| 29. Quotations presented Were participant quotations presented to illustrate the themes / findings? Was each  quotation identified? e.g. participant number | Results, p. 11-16 |
| 30. Data and findings consistent Was there consistency between the data presented and the findings? | Results, p. 11-16 |
| 31. Clarity of major themes Were major themes clearly presented in the findings? | Results, p. 11-16 |
| 32. Clarity of minor themes Is there a description of diverse cases or discussion of minor themes? | Results, p. 11-16f |

From: Tong A, Sainsbury P, Craig J. Consolidated criteria for reporting qualitative research (COREQ): a 32-item checklist for interviews and focus groups. Int J Qual Health Care. 2007;19(6):349-357.
