# Supplementary material for: Hospital pharmacists’ experiences of participating in a partnered pharmacist medication charting credentialing program: a qualitative study
Source: BMC Health Serv Res. 2021 Mar 19;21:251. doi: 10.1186/s12913-021-06267-w (PMC7980669; doi:10.1186/s12913-021-06267-w)
Supplement: Supplementary file 2 — Additional file 2: Supplementary File 2. Interview guide for health professionals and key informants [file 12913_2021_6267_MOESM2_ESM.docx]

**Supplementary File 2. Interview guide for health professionals and key informants**

Q1. What is your understanding of the changes that are about to occur (please describe how processed have changed as a result of the model)? Before and after change:

- Who is involved? (patient, physician, different nursing staff etc.)
- Why is it done this way? (organisational issues)
- What was/is your contribution to the safety and quality of care provided under the old/new model?
- How would you describe the team dynamics before the model was implemented?
- What was the impact if the new model?

Q.2 How do you feel about these changes? (prompts – relative advantage, trialability, simplicity, observability of results, compatibility with existing values and practices)

- How satisfied were you before/how satisfied are you now with the new model?
- What do you think about the support and training and offer? (ask specifically about the value of the credentialing process and if ongoing credentialing is important?)
- How has this affected your clinical competencies and ability to apply your skills optimally?
- What is the impact of the new model on safety and quality of care?
- What has been the impact of the new model upon your workload? When it comes to medication charting, does the new model impose more work on you or less, or about the same?

Q.3 How do you think this new model will work out if rolled out across the hospital?

- Will the workforce have adequate capacity
- How has it impacted on the time available for you to do your work? [more efficient? Impact on administration load? What did they do with any time they have saved?]
- How well would this model work in other hospitals?

Q.4 What are the factors that (will) impact on your ability to implement this system effectively? (patient, hospital, clinician, external organisation level)

- Profession-specific interviews only: How do the different health professionals work together to ensure that accurate medication lists are achieved? (pre- and post- implementation)
- Do you see any problems or strengths about this model (for your hospital/generally)?
- What are the risks associated with this model?
